# A foetus with 18p11.32-q21.2 duplication and Xp22.33-p11.1 deletion derived from a maternal reciprocal translocation t(X;18)(q13;q21.3)

**DOI:** 10.1186/s13039-018-0381-5

**Published:** 2018-06-13

**Authors:** Jun-Kun Chen, Ping Liu, Li-Qin Hu, Qing Xie, Quan-Fei Huang, Hai-Liang Liu

**Affiliations:** 1 Medical Genetic Centre of Ganzhou Maternal and Child Health Care Hospital, Ganzhou, 341000 China; 2 CapitalBio Genomics Co., Ltd., Dongguan, 532808 China

**Keywords:** The non-invasive prenatal testing (NIPT), Sub-chromosomal abnormalities, Unbalanced translocation, X-chromosome inactivation

## Abstract

**Background:**

Non-invasive prenatal testing (NIPT) evaluates circulating cell-free DNA (cfDNA) and has been widely applied, with highly accurate results for detecting foetal trisomies 21, 18 and 13. Recently, increasing attention has been paid to the clinical application of the non-invasive detection of foetal sub-chromosomal duplications and deletions beyond common aneuploidies.

**Case presentation:**

A 32-year-old healthy pregnant woman was referred to the Medical Genetic Centre of Ganzhou Maternal and Child Health Care Hospital. As routine practice, ultrasound examination at a gestational age of 16 weeks showed that the foetus is normal. To avoid invasive prenatal diagnosis procedures, an NIPT was offered to further screen for common foetal chromosomal abnormalities. The result showed that there was an approximately 50.94 Mb duplication in p11.32-q21.2 of chromosome 18 and an approximately 58.46 Mb deletion in p22.33-p11.1 of chromosome X. In addition, the chromosome karyotypes of the parents and foetus were also analysed. Chromosome karyotype analysis results showed that foetal karyotype was 46,X,der(18), the maternal karyotype was 46,XX,t(X;18)(q13;q21.3), and the paternal karyotype revealed no obvious abnormality.

**Conclusion:**

In this case, we successfully detected a healthy pregnant woman with balanced translocation X;18(q13;q21.3) and described the foetal karyotype as 46,X,der(18)t(X;18)(q11;q21.1)mat. Our report illustrated these cases which present complex X;autosome balance translocation and X;autosome unbalance translocation which may contribute to severe clinical phenotypes. In addition, our report also proved that the interruption of genes in the Xq critical region is not only reason of primary infertility. Finally, we prompted that NIPT might play a role in the first trimester screening of sub-chromosomal rearrangement.

## Background

Non-invasive prenatal testing (NIPT) for common foetal aneuploidies has been widely applied, with highly accurate results for detecting foetal trisomies 21, 18 and 13 [1]. The specificity and sensitivity of these tests can reach 99% [2]. Recently, increasing attention has been paid to the clinical application of the noninvasive detection of foetal sub-chromosomal duplications and deletions beyond common aneuploidies [3, 4].

Balanced reciprocal chromosomal translocations, which occur when there is an exchange of terminal segments between different chromosomes, are relatively common genetic abnormalities. The incidence of balanced reciprocal translocations ranges from approximately 1/800 to 1/1100, affecting 35.5 of every 1000 couples with recurrent pregnancy loss [5]. Women with balanced X-autosome translocations are a rare and clinically heterogeneous group of patients whose most common phenotype is infertility [6, 7]. The consequences of balanced X-autosome translocations in female carriers depend mostly on the location of the breakpoints and on the X-chromosome inactivation (XCI) pattern [8]. The mechanism of XCI is not completely known. Several studies in mice demonstrated that the X-chromosome inactivation centre (Xic)-a single *cis*-acting control locus at Xq13.2 that is necessary for: (1) initiation of XCI by counting the number of X chromosomes and choosing one X chromosome to be inactivated; (2) spreading the inactivation of the X chromosome in the *cis* position; and (3) maintaining the inactive state [9].

Recently, studies on women with rearrangements involving X-chromosomes indicate that breakpoints in patients with premature ovarian failure are usually concentrated in the long arm of the X chromosome [7, 10], in a specific region that spans from Xq13 to Xq27 called the “Xq critical region,” which is assumed to be responsible for the maintenance of ovarian function and normal reproductive lifespan.

Here, we reported one case of a 32-year-old healthy pregnant woman with balanced translocation X;18(q13;q21.3) and the foetus with abnormal NIPT indicating duplication in chr18 and deletion in chrX. Combined with the karyotype analysis results of the parents and foetus, the genetic abnormalities in the foetus were derived from a balanced translocation of 46,XX,t(X;18)(q13;q21.3) in the mother. This case suggested that NIPT is a valuable method for screening genetic abnormalities derived from parents with balanced translocations in the first trimester screening and demonstrate balanced Xq-autosome translocations is not the cause of infertility.

## Case Presentation

A 32-year-old healthy pregnant woman was referred to the Medical Genetic Centre of Ganzhou Maternal and Child Health Care Hospital. The pregnant woman was 160 cm tall and weighed 53 kg with normal hallmark developmental milestones. She delivered a boy 10 years ago. During the second trimester maternal serum screening in another hospital, she was notified that the foetus had an increased risk of developing T18 syndrome. The study participant was at 16 weeks’ gestation. As is routine practice, an ultrasound was conducted to monitor the developmental status of the foetus. The ultrasound examination at a gestational age of 16 weeks showed that the foetus was normal. To avoid invasive prenatal diagnosis procedures, an NIPT was offered to further screen for common foetal chromosomal abnormalities. This project was approved by the Research Ethics Committee of Ganzhou Maternal and Child Health Care Hospital. Written informed consent was obtained from all of the participants or guardians that participated in this research.

## Materials and methods

### Non-invasive prenatal testing

Experiments and data analysis were performed using the technology platform provided by CapitalBio Genomics Co., Ltd. 10 mL of peripheral blood was collected from the pregnant woman in EDTA containing tubes or streck blood collection tubes. The peripheral blood samples were first centrifuged at 1600×g for 10 min at 4 °C to separate the plasma from blood cells. The plasma portion was carefully transferred to a polypropylene tube and subjected to centrifugation at 16,000×g for 10 min at 4 °C to pellet the remaining cells [11]. Cell-free foetal DNA (cffDNA) from 600 μL of maternal plasma was extracted using the QIAamp DSP DNA Blood Mini Kit (Qiagen) following the blood and body fluid protocol. For the semiconductor sequencing platform, DNA from maternal plasma was used for library construction according to the Ion Plus Fragment Library Kit (Life Technologies, America), and semiconductor sequencing was performed using an Ion Proton sequencer (Life Technologies, America) at 400 flows according to the manufacturer’s instructions. Based on our previous study, we developed a technique that uses the read length to estimate the concentration of foetal Cell free DNA (cfDNA) in maternal plasma by sequencing [2]. The retained reads were aligned to the human genomic reference sequences (hg19) using the BWA. The foetal DNA concentration was calculated as a quality control, as described in Yin’s paper [12]. Combined GC-correction and Z-score testing methods were used to identify foetal autosomal aneuploidy for trisomy, as described in Liao’s paper [2]. Z scores ranged from − 3 to 3 and were considered to indicate low risk for a trisomy chromosome.

### Chromosome karyotype analysis

Chromosome karyotype analyses with under sterile conditions, was performed for the foetus and parents, on cultured amniocytes and lymphocytes according to standard protocols. The amniocentesis was performed with the guidance of ultrasound and centrifuged, inoculated in the culture medium and cultured under 37 °C. Once many circular translucent dividing cells had emerged, colchicine was added and cultured for another three hours. When the number of circular translucent cells increased, cells were harvested for chromosome preparation. Subsequently, 3 mL of parents’ peripheral blood were collected with heparin anticoagulation and inoculated in the phytohemagglutinin (PHA) culture medium for further karyotype analysis. According to the principle of “An International System for Human Cytogenetic Nomenclature, ISCN2013”, a total of 60 dividing phases were counted using an AI chromosome image analysis system (CytoVision, Switzerland), 20 karyotypes were analysed and repeated 3 times.

## Results

### Non-invasive prenatal testing

**Fig. 1:**
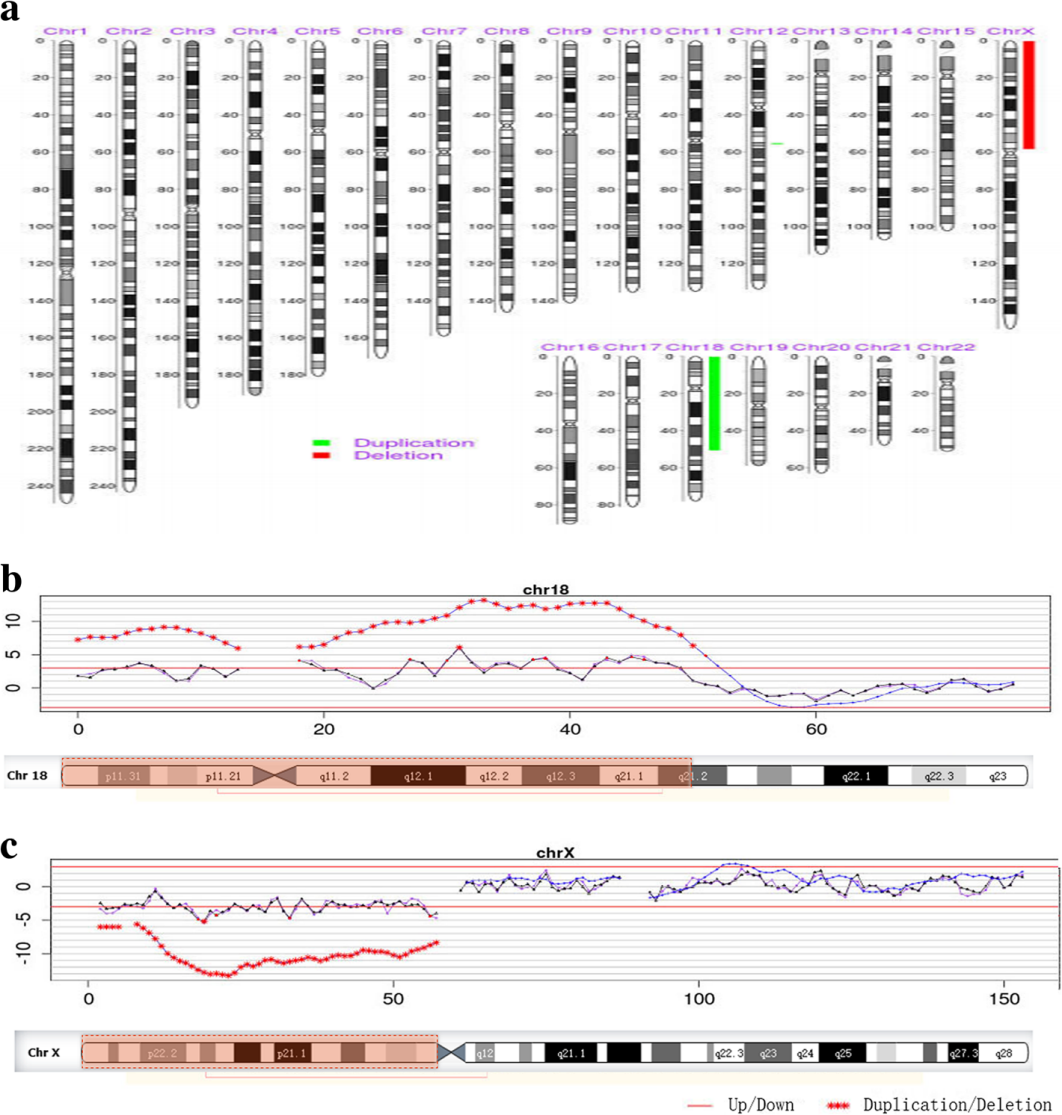
NIPT results for the foetus. **a** Analysis result of foetal karyotype obtained by NIPT. Among them, red stripe directed deletion part of chrX and green stripe directed duplication part of chr18; **b** Approximately 50.94 Mb duplications in p11.32-q21.2 of chromosome 18; **c** Approximately 58.46 Mb deletions in p22.33-p11.1 of chromosome X

The NIPT result showed that the Z scores of chromosomes 18 and X were 10.152 and − 2.808, respectively; these scores suggested that duplication/deletion of foetal DNA fragments occur in chromosomes 18 and X. Consequently, the method for the detection of foetal chromosomal duplications/deletions, as previously described [2], demonstrated an approximately 50.94 Mb duplication in p11.32-q21.2 of chromosome 18 and an approximately 58.46 Mb deletion in p22.33-p11.1 of chromosome X (Fig. 1a, b, c).

### Chromosome karyotype analysis

**Fig. 2:**
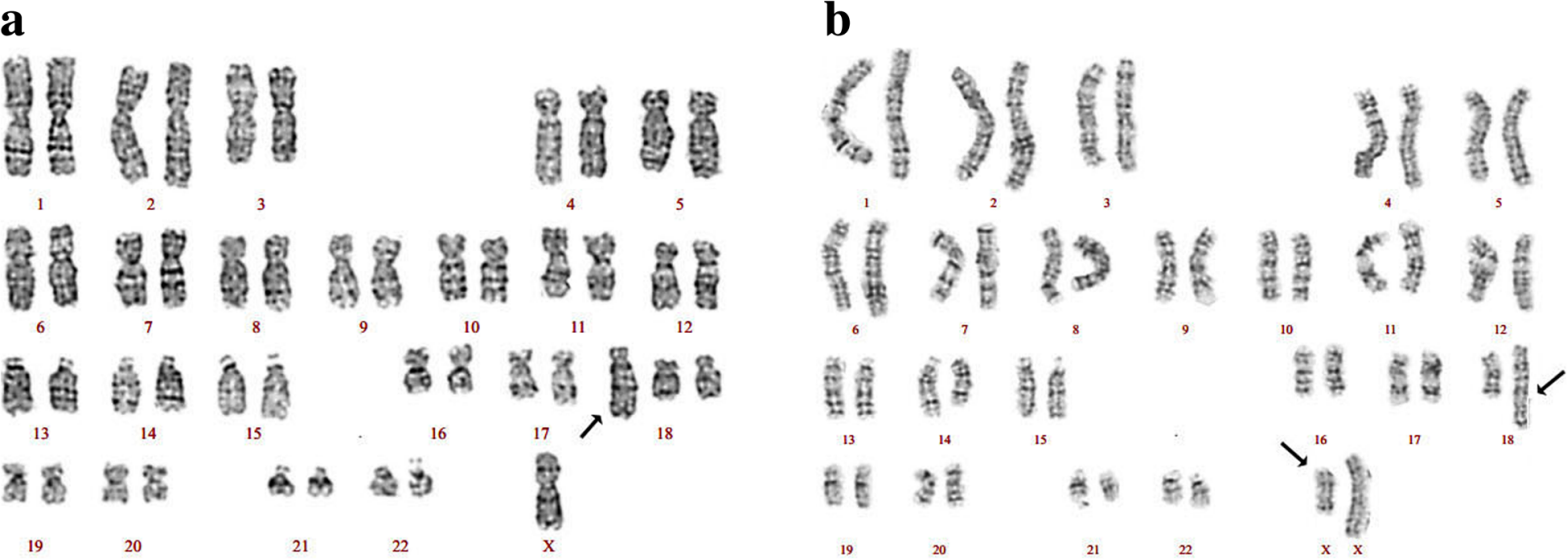
GTG banding results for pregnancy women and feotus. **a** The foetal karyotype was 46,X,der(18); **b** The maternal karyotype was 46,XX,t(X;18)(q13;q21.3). Black arrow directed the abnormal chromosome

Karyotype analysis of amniotic fluid showed chromosome structural abnormalities 46,X,der(18) (Fig. 2a), although the precise chromosomal translocation section was unknown. Moreover, we also analysed the chromosome karyotypes of the parents; the maternal karyotype 46,XX,t(X;18)(q13;q21.3) showed balanced translocation of chromosomes 18 and X (Fig. 2b), while the paternal karyotype revealed no obvious abnormality.

## Discussion

In this report, NIPT indicated foetal chromosomal abnormalities with duplications in chr18 and deletions in chrX prior to ultrasound and invasive prenatal testing. NIPT has the advantages of noninvasive, early and high throughput, as well as high accuracy, it was not only suitable for the detection of the traditional chromosome aneuploidy, but also for complex fetal subchromosome structure aberrations and copy number variations. Although routine NIPT has been used increasingly in clinical practice, but it is still a screening test, follow-up diagnosis is needed to confirm accurate chromosome structural abnormalities and the parental origin.

In the present study, the maternal karyotype 46,XX,t(X;18)(q13;q21.3) showed balanced translocation of chromosomes 18 and X. The X;autosome balance translocation in this region was first discovered. Translocations involving gonosomes are rare and appear to contribute significantly to primary infertility. X;autosome translocations occur in approximately 1/30,000 live births and may show different breakpoints on the X chromosome [13]. X-chromosomal activation/inactivation combined with variable gene expression can occur because translocated genetic material from the X-chromosome may or may not be reactivated once translocated [14]. In balanced X;autosome translocations, the normal X chromosome is usually, though not always, inactivated to prevent deleterious monosomy of autosomal genes. In addition, the inactivation of the derivative X chromosome will spread into the translocated autosomal sequences resulting in a silencing of the autosomal genes [15]. In this case, the pregnant woman points to the hypothesis that the interruption of genes in the Xq critical region may not the only reason for primary infertility. Currently, several other hypotheses have been considered, most of them related to an effect position mechanism altering gene expression in the X-chromosome breakpoint flanking regions or in the autosomes involved in the rearrangements. Investigation and ongoing analysis of such anomalies may explain the often idiopathic nature of primary infertility syndromes.

In this case, we are the first to report this type of unbalanced translocation in the foetus. The duplications in p11.32-q21.2 of chromosome 18 in the foetus involves “trisomy 18 syndrome”, also known as Edward’s syndrome, which is the second most common trisomy syndrome [16]. Edward’s syndrome, as a genetic disorder, is caused by full, mosaic, partial trisomy 18q or partial trisomy 18p [17]. 18q partial trisomy was first reported by Cohen et al. [18] in 1972, while nearly 40 cases [19] have now been reported. Most 18q partial trisomy cases inherited from parents are balanced rearrangements [2, 20, 21]. It has been reported in the literature that a partial deletion of Xp may arise de novo, or as the result of parental balanced translocations involving a segment of the short arm of chromosome X [22–24]. The Online Mendelian Inheritance in Man (OMIM) database was searched regarding the missing area, Gillard et al. [25] used a RFLP closely linked to Steroid sulphatase deficiency to demonstrate deletion of the Steroid sulphatase deficiency locus at Xpter-p22.3 in 8 of 9 families with X-linked ichthyosis. For this condition, the severity of the symptoms and the phenotype is highly variable depending on the extension of chromosome involvement and the level of compromised cells and tissues. In our opinion, it will lead to birth defects if the foetus is born.

## Conclusion

In conclusion, combining NIPT and karyotype analysis, we successfully detected a healthy pregnant woman with balanced translocation X;18(q13;q21.3) and a foetal karyotype of 46,X,der(18)t(X;18)(q11;q21.1)mat. Either the mother’s balanced translocation or the foetus’s unbalanced translocation was detected. Our report illustrated these cases which present complex X;autosome balance translocation and X;autosome unbalance translocation which may contribute to severe clinical phenotypes. Moreover, our report also proved that the interruption of genes in the Xq critical region is not the only reason of primary infertility. The rare and complex phenotypes should be investigated to define the subsets and allow phenotypic classification. Finally, we prompted that NIPT might play a role in the first trimester screening of sub-chromosomal rearrangement.

## Abbreviations

cfDNA: Cell free DNA
cffDNA: Cell-free fetal DNA
NIPT: Noninvasive prenatal testing
OMIM: Online Mendelian Inheritance in Man
T18: Trisomy 18
XCI: X-chromosome inactivation
Xic: X-chromosome inactivation center
